# Objective Determination of End of MERS Outbreak, South Korea, 2015

**DOI:** 10.3201/eid2201.151383

**Published:** 2016-01

**Authors:** Hiroshi Nishiura, Yuichiro Miyamatsu, Kenji Mizumoto

**Affiliations:** The University of Tokyo, Tokyo, Japan (H. Nishiura, Y. Miyamatsu, Kenji Mizumoto);; Japan Science and Technology Agency, Kawaguchi Saitama, Japan (H. Nishiura, Y. Miyamatsu, K. Mizumoto)

**Keywords:** MERS, Middle East respiratory syndrome, coronavirus, outbreak, risk assessment, mathematical model, epidemiology, South Korea, viruses, zoonoses

**To the Editor:** After not finding any additional cases of Middle East respiratory syndrome (MERS) for several weeks in South Korea, in July 2015, the South Korean government and the World Health Organization (WHO) discussed the appropriate time to declare the end of the outbreak in July 2015 (*1*). This declaration would enable allocation of human resources to healthcare facilities to return to normal and would help restore international travel to the country. A widely acknowledged criteria of WHO to determine the end of an epidemic has been twice the length of the incubation period since the most recently diagnosed case (*2*). For MERS, the longest incubation period is 14 days. Thus, adopting 28 days as the waiting period, and counting days from diagnosis of the most recent case on July 4, 2015, the earliest date the South Korean government could have declared the end of outbreak was August 2 if it adhered to WHO criteria (*1*). However, to emphasize safety to the nation and to international travelers at an earlier time, the South Korean government originally decided to announce the end of the MERS outbreak on July 27, the date the last quarantined MERS patient was released from movement restriction. Because we are concerned about the validity of strict adherence to the WHO criteria, we objectively calculated the probability of observing additional cases at a given time and compared that probability with the WHO criteria.

To clearly define the end of the outbreak, we excluded reintroduction of imported cases and cases of MERS coronavirus infection resulting from a zoonotic reservoir. We defined the end of the outbreak as the end of continued chains of transmission. The probability of observing additional cases was derived by using the serial interval; that is, the time from illness onset in the primary case-patient to illness onset in a secondary case-patient, and the transmissibility of MERS (Technical Appendix). Both of these epidemiologic variables were estimated by using case data in South Korea (*3*,*4*). As practiced in the determination of the length of quarantine (*5*), the end of outbreak can be declared if that probability is <5%, a threshold value.

Our analysis showed that the first date on which the posterior median probability decreased to <5% was July 21 (Figure, panel A). The first date on which the posterior median decreased to 1% was July 23. Compared with August 2 as calculated from the WHO criteria, the end of the outbreak could have been declared 11 and 9 days earlier, respectively. Because the choice of 5% or 1% as the threshold probability is arbitrary (as practiced in determining the p value in any hypothesis testing) and because of the need to account for parameter uncertainties, we also measured the sensitivity of the first date on which the South Korean government could declare the end of the outbreak to a variety of threshold values (Figure, panel B). Examination of the probability of observing additional cases in the range of 0.5% to 10% indicated the end of the outbreak could have been declared from July 21 to July 24 (i.e., 9–12 days earlier than August 2).

**Figure:**
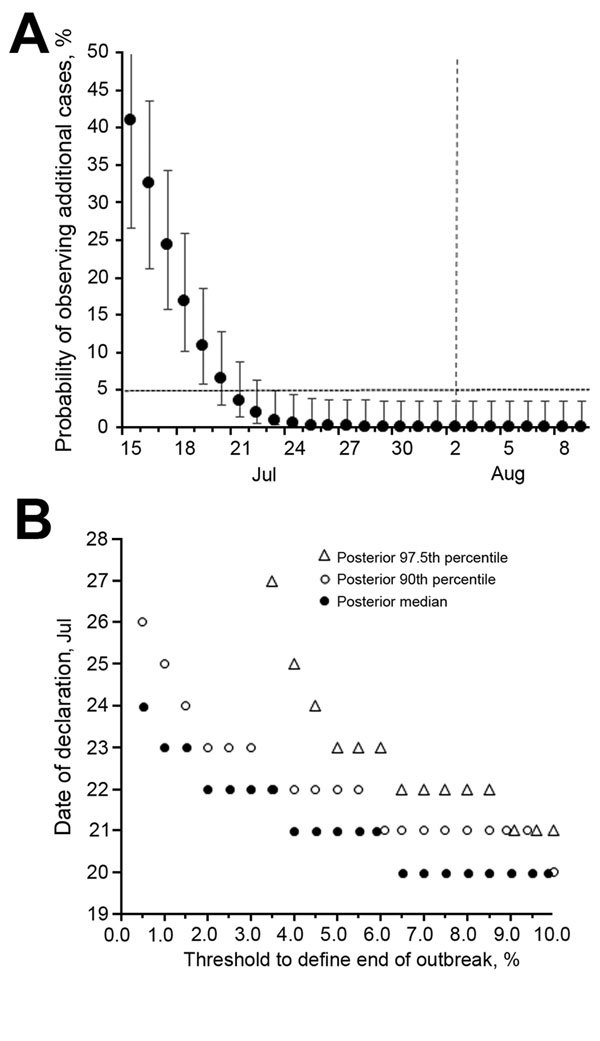
Estimated probability of observing additional cases of Middle East respiratory syndrome coronavirus infection, South Korea, 2015. A) Estimated probability of observing additional cases on each calendar date, given that no illness onset has been observed by the specified date. Circles represent posterior median values; whiskers extend to upper and lower 95% credible intervals. Horizontal dashed line represents 5%, a threshold level. Vertical line indicates August 2, 2015, on which the end of the outbreak might be declared if World Health Organization criteria were followed. B) Calendar date to declare the end of outbreak for different threshold probabilities and percentile points of posterior distribution. Horizontal axis corresponds to the probability of observing additional cases. Vertical axis shows the date of declaration which is calculated as 1 day plus the date at which the probability of observing additional cases lowered the specified threshold probability.

Our proposed method does not account for missing undiagnosed or mild cases, and underdiagnosis would considerably extend the time to declare the end of an outbreak (and thus the proposed method is not directly applicable to, for example, Ebola virus disease in West Africa, for which we are currently developing an alternative method). All possible contact with diagnosed case-patients in the late phase of the MERS outbreak in South Korea were traced (*6**,**7*); thus, we believe it was appropriate to ignore ascertainment bias in this specific setting. Although our proposed approach is simplistic, adopting the WHO criteria could have added >1 week to the elevated state of tension, and the use of the incubation period distribution would be fully supported only when the exact times of infection were known for exposed potential contacts. Although it is a posteriori reasoning, the original decision made by the South Korean government at an earlier date was ironically supported by our proposed method. Rather than adopting the use of “twice” and the “incubation period,” which has not been theoretically justified, an objective decision of the end of an outbreak should explicitly rest on the risk of observing at least 1 more case on or after a specified date.

Technical Appendix Epidemiologic data, probabilistic model, and supplementary discussion.
