# Supplementary material for: Objective Determination of End of MERS Outbreak, South Korea, 2015
Source: Emerg Infect Dis. 2016 Jan;22(1):146–8. doi: 10.3201/eid2201.151383 (PMC4696716; doi:10.3201/eid2201.151383)
Supplement: Technical Appendix — Epidemiologic data, probabilistic model, and supplementary discussion. [file 15-1383-Techapp-s1.pdf]

Article DOI: http://dx.doi.org/10.3201/eid2201.151383

Objective Determination of End of MERS Outbreak, South Korea, 2015

Technical Appendix

Epidemiologic Data

We analyzed the date of illness onset among persons who had laboratory-confirmed Middle East respiratory syndrome (MERS) cases in South Korea during 2015 (*1*–*3*). The latest date on which the data were compiled was October, 1, 2015 with a total of 185 confirmed cases in South Korea (excluding 1 case in a person who traveled overseas). Whenever the date of illness onset was missing, we substituted it with the date of laboratory confirmation. In total, there have been 4 cases with unknown dates of illness onset since June 15, but this substitution actually enabled us to conservatively argue the time to declare the end of outbreak: counting the waiting period from the date of diagnosis elevates the actual probability of the freedom from infection (*4*). Considering that illness developed in 2 persons on July 2, the end of MERS outbreak could be declared on July 31, at the earliest, following the WHO criteria. Nevertheless, to be conservative with the WHO method, counting the diagnosis date of 1 of the latest cases, July 4, as the first day, the earliest date to declare the end of outbreak may be August 2 (*5*).

To propose a more objective approach, 2 pieces of epidemiologic information were used. First, we used the distribution of the serial interval, i.e., the time from illness onset of a primary case to illness onset of the secondary case in a person who directly acquired infection from the primary case-patient (*6*). An analysis of epidemiologic data in South Korea estimated the mean

and SD of the serial interval at 12.6 and 2.8 days, respectively (*3*). In the following, the cumulative distribution function of the serial interval is denoted by $F(t)$ which was assumed to follow a gamma distribution. Second, we used parameters that govern the transmissibility of MERS, i.e., R_0 at 0.75 and dispersion parameter k at 0.14 of a negative binomial distribution (*7*), which did not significantly deviate from published estimates in earlier studies (*8*,*9*). By using the estimated reproduction number and dispersion parameter, the cluster size of >150 cases was not unexpected (*10*). These parameters are applicable to an initial exponential growth phase without interventions, and not specifically applicable to a nonlinear phase under contact tracing practice. Thus, it should be remembered that the use of these parameters would lead to an overestimation of the probability of observing additional cases, and thus, the proposed approach is deemed conservative. To address parameter uncertainties, we used a bivariate normal distribution that accounts for parameter dependence and resampled randomly drawn combinations of R_0 and k as practiced before (*7*). The similar resampling was conducted for the serial interval distribution (i.e., the mean and the standard deviation) (*3*).

Probabilistic Model

Here we devise a model that calculates the probability of observing additional cases at a given calendar time, counting waiting time from dates of illness onset in potential primary cases. If 1 minus this probability is >95% on a given date, one can be 95% sure that the outbreak is over by that date. For simplicity, we ignore potential asymptomatic infection with MERS coronavirus in the following analysis.

If we were concerned with only a single (potential) secondary case, the probability that an outbreak is over at t days since the date of illness onset in the potential primary case would be

given by $F(t)$ (*11*). Nevertheless, an appropriate computation of the probability of observing additional cases on a calendar date involves 3 major obstacles that require some improvements. First, there can be multiple cases on the latest date, which was actually the situation for the MERS outbreak in South Korea. If there are 2 cases, the absence of transmission will be calculated at $(F(t))^2$, assuming that 2 cases independently produce secondary cases, and thus, the probability of observing at least 1 case is calculated as $1-(F(t))^2$. If there are n cases, the probability of observing additional cases is obtained as $1-(F(t))^n$. Second, there can be several persons who developed the illness on different dates around the latest time. Suppose that the days elapsed from 2 persons with different dates of illness onset were t_1 and t_2, the probability of observing additional cases will have to be calculated as $1-F(t_1)F(t_2)$. Third, we have to address the potential for observing multiple secondary cases produced by a single primary case, e.g., multiple infections among healthcare workers who were exposed to an admitted patient. The variation in the number of secondary cases per single primary case can be addressed by using both R_0 and k, which could even partially capture the emergence of superspreaders. Let p_y be the probability that the number of secondary cases is y, i.e., $p_y = \Pr(Y = y)$. Using the dataset of t_i, the calendar date of illness onset of diagnosed cases i (i = 0,1,..,185), the probability of observing additional cases in future at calendar date t is calculated as

$$\Pr(one\ or\ more\ cases) = 1 - \prod_{i=1}^{185} \sum_{y=0}^{\infty} p_y [F(t - t_i)]^y$$

(Equation 1)

Equation 1 does not manually subtract all existing secondary transmissions from the model, despite the fact that the observed cases have already generated secondary cases that they were supposed to cause. For that reason, the probability that is derived from the Equation 1 might

be a slight overestimate. Nevertheless, to keep the model structure simple, we let the model to be simple as shown and conservative. At least, observed cases for which illness recently developed did not involve superspreaders (and the bias introduced by the above-mentioned model assumption would be minimal). The simulations with resampled serial interval (mean and SD), R_0 and k were run 10,000 times, enabling us to take percentile points for the calculation of uncertainty bounds.

Supplementary Discussion

The calculated probability is interpreted as the risk of observing at least 1 more case on or after a specified date and has a good potential to assist objective determination of the end of outbreak. The model efficiently addressed 3 practical problems in objectively calculating the probability that an outbreak leads to the end: 1) multiple cases on the latest date, 2) several recent cases with different illness onset dates, and 3) variations in the number of secondary cases generated by a single primary case.

The cutoff probability is arbitrarily determined, as practiced to determine the length of quarantine period using the incubation period (*4*). Despite arbitrariness, p value in all hypothesis testing is determined in the same fashion. Rather than the issue of adopting a specific threshold probability, the point of devising the proposed model is to explicitly calculate the probability of observing additional cases at a given point in time. Relying on the use of the incubation period can be feasible only when the exact time of exposure is known for all traced contacts, but such situation is usually not the case for directly transmitted diseases, and thus, one should remember that the incubation period is applicable to specific settings with known times of exposure among all potential contacts.

References

1. World Health Organization. Global Alert and Response (GAR). Coronavirus infections [cited 2015 Jul 15]. http://www.who.int/csr/don/archive/disease/coronavirus_infections/en/

2. Republic of Korea Ministry of Health & Welfare. As of 10:00 15 June [cited 2015 Jul 1]. http://www.mw.go.kr/front_new/al/sal0301ls.jsp?PAR_MENU_ID=04&MENU_ID=0403

3. Cowling BJ, Park M, Fang VJ, Wu P, Leung GM, Wu JT. Preliminary epidemiological assessment of MERS-CoV outbreak in South Korea, May to June 2015. Euro Surveill. 2015;20:7–13. PubMed

4. Nishiura H. Determination of the appropriate quarantine period following smallpox exposure: an objective approach using the incubation period distribution. Int J Hyg Environ Health. 2009;212:97–104. PubMed http://dx.doi.org/10.1016/j.ijheh.2007.10.003

5. The Chosunilbo. Official end to MERS outbreak expected next month [cited 2015 Jul 15]. http://english.chosun.com/site/data/html_dir/2015/07/13/2015071301669.html

6. Fine PE. The interval between successive cases of an infectious disease. Am J Epidemiol. 2003;158:1039–47. PubMed http://dx.doi.org/10.1093/aje/kwg251

7. Nishiura H, Miyamatsu Y, Chowell G, Saitoh M. Assessing the risk of observing multiple generations of Middle East respiratory syndrome (MERS) cases given an imported case. Euro Surveill. 2015;20:21181. PubMed

8. Breban R, Riou J, Fontanet A. Interhuman transmissibility of Middle East respiratory syndrome coronavirus: estimation of pandemic risk. Lancet. 2013;382:694–9. PubMed http://dx.doi.org/10.1016/S0140-6736(13)61492-0

9. Cauchemez S, Fraser C, Van Kerkhove MD, Donnelly CA, Riley S, Rambaut A, et al. Middle East respiratory syndrome coronavirus: quantification of the extent of the epidemic, surveillance biases,

and transmissibility. Lancet Infect Dis. 2014;14:50–6. PubMed http://dx.doi.org/10.1016/S1473-3099(13)70304-9

10. Kucharski AJ, Althaus CL. The role of superspreading in Middle East respiratory syndrome coronavirus (MERS-CoV) transmission. Euro Surveill. 2015;20:14–6. PubMed

11. Nishiura H, Wilson N, Baker MG. Quarantine for pandemic influenza control at the borders of small island nations. BMC Infect Dis. 2009;9:27. PubMed http://dx.doi.org/10.1186/1471-2334-9-27
